# Adenylate Cyclase Toxin Promotes Internalisation of Integrins and Raft Components and Decreases Macrophage Adhesion Capacity

**DOI:** 10.1371/journal.pone.0017383

**Published:** 2011-02-23

**Authors:** César Martín, Kepa B. Uribe, Geraxane Gómez-Bilbao, Helena Ostolaza

**Affiliations:** Unidad de Biofísica and Departamento de Bioquímica, Universidad del País Vasco, Bilbao, Spain; French National Centre for Scientific Research - Université de Toulouse, France

## Abstract

*Bordetella pertussis*, the bacterium that causes whooping cough, secretes an adenylate cyclase toxin (ACT) that must be post-translationally palmitoylated in the bacterium cytosol to be active. The toxin targets phagocytes expressing the CD11b/CD18 integrin receptor. It delivers a catalytic adenylate cyclase domain into the target cell cytosol producing a rapid increase of intracellular cAMP concentration that suppresses bactericidal functions of the phagocyte. ACT also induces calcium fluxes into target cells. Biochemical, biophysical and cell biology approaches have been applied here to show evidence that ACT and integrin molecules, along with other raft components, are rapidly internalized by the macrophages in a toxin-induced calcium rise-dependent process. The toxin-triggered internalisation events occur through two different routes of entry, chlorpromazine-sensitive receptor-mediated endocytosis and clathrin-independent internalisation, maybe acting in parallel. ACT locates into raft-like domains, and is internalised, also in cells devoid of receptor. Altogether our results suggest that adenylate cyclase toxin, and maybe other homologous pathogenic toxins from the RTX (Repeats in Toxin) family to which ACT belongs, may be endowed with an intrinsic capacity to, directly and efficiently, insert into raft-like domains, promoting there its multiple activities. One direct consequence of the integrin removal from the cell surface of the macrophages is the hampering of their adhesion ability, a fundamental property in the immune response of the leukocytes that could be instrumental in the pathogenesis of *Bordetella pertussis*.

## Introduction

Adenylate cyclase toxin (ACT) is an essential virulence factor secreted by *Bordetella pertussis*, the bacterium that causes whooping cough [1]. This severe childhood disease remains endemic worldwide despite extensive vaccination programmes [2]. ACT is a ≈200 kDa calmodulin-activated adenylyl cyclase toxin [3]–[5] that behaves as an anti-inflammatory and anti-phagocytic factor, facilitating colonization of the respiratory tract by *B. pertussis* [2], [6]. Upon binding to its cell surface receptor, the α_M_β_2_ integrin [7], ACT becomes an integral membrane protein and inserts its N-terminal adenylyl cyclase domain (AC domain) into the cytosol of the target cell. After binding to calmodulin, ACT–AC raises the intracellular cAMP concentration in host cells cAMP to a pathological level [8], [9]. In addition, ACT can form cation-selective small pores, independent of AC domain translocation, which permeabilise cell membranes at high toxin concentrations [10], [11].

More recently, the toxin has been shown to induce rises in intracellular [Ca^2+^] in target cells [12], [13]. Elevation and modulation of free cytosolic calcium concentrations by bacterial toxins has been described as one of the basic strategies of host cell manipulation by pathogens. By inducing Ca^2+^ signalling, some bacterial toxins can induce the expression and secretion of pro-inflammatory mediators. Bacteria can also induce Ca^2+^ responses that play a role in the cytoskeletal rearrangements required for cell binding and for internalisation of the microorganism [14].

ACT is a member of the RTX (Repeats-in-Toxin) family of proteins that share a characteristic calcium-binding motif of Gly- and Asp-rich nonapeptide repeats, and marked cytolytic or cytotoxic activity [9], [15]. Like other members of this family, the mature form of ACT is fatty-acylated. First produced as an inactive protoxin, pro-ACT, it is then converted to an active toxin by post-translational palmitoylation of an internal lysine (Lys 983), a process catalyzed by a dedicated acyltransferase, CyaC [16]. Acylation, especially covalent linking of saturated fatty acids, represents a targeting signal for many proteins that interact with membrane microdomains [17]. The requirement of lipid microdomains for the cytotoxity induced by various RTX toxins, particularly leukotoxins from *Mannheimia haemolytica* and *Actinobacillus actinomycetemcomitans,* has been pointed out in the last few years [18], [19]. Binding of proteins to lipid rafts may result in internalisation of such proteins into cells. There are many examples of bacterial toxins, pathogenic bacteria and viruses that use lipid rafts and raft-associated caveolae to bind to cells and induce their internalisation [20], [21].

Membrane rafts are currently considered to consist of transient nanoscopic domains enriched in sphingolipids and cholesterol and have a characteristic protein composition and physicochemical properties different from the surrounding bulk membrane [20], [21]. Accumulating evidence suggests that these domains play important roles in cellular functions such as membrane trafficking, endocytosis, cell adhesion mechanisms and regulation of signalling pathways [22]. Numerous pathogenic bacteria, bacterial toxins and viruses have been reported to use rafts or raft-like membrane domains (RLMDs) as cell surface platforms to interact, bind and possibly enter host cells [23]–[25].

Toxins that use lipid rafts as part of their virulence strategy have receptors that are raft components [26], [27]. However, ACT binds to host cells through the integrin CD11b/CD18 receptor, which does not associate with lipid rafts before cell activation has taken place [28]. While inactive, β_2_ integrins are confined to non-RLDM locations due to their anchorage to cytoskeletal proteins such as talin [28], [29]. One mechanism that allows the movement of integrins into RLMDs involves the calcium-dependent activation of calpain, a protease that hydrolyzes talin, releasing integrins from their anchoring to the cytoskeleton [28], [29]. Very recently, such a mechanism has been reported to be involved in the recruitment of ACT - CD11b/CD18 integrin complexes into membrane rafts promoted by toxin-induced calcium influx [30].

In view of recent data from our laboratory showing that ACT induces increases in [Ca^2+^]_i_ in target cells [13], we designed this research to explore the downstream effects derived from this toxin-induced calcium influx. In particular, we have addressed its implication in possible toxin-induced internalisation processes. We show here that ACT and integrin molecules, along with other raft components, are rapidly internalized by the macrophages in a toxin-induced calcium rise-dependent process, affecting the adhesion properties of these immune cells. The removal of domains that contain key molecules such as integrins, and perhaps other important signalling molecules, from the leukocyte plasma membrane may represent a beneficial strategy followed by pathogenic *Bordetella* to circumvent the host immune system.

## Results

### ACT is internalised and promotes the internalisation of integrins and membrane raft domains in J774A.1 macrophages

Bacteria can induce Ca^2+^ responses that play a role in cytoskeletal rearrangements required for cell binding and for internalisation of the microorganism [14]. Further, bacterial toxins, pathogenic bacteria and viruses that use lipid rafts and raft-associated caveolae to bind to cells can induce internalisation of the pathogen [26], [31]. In the last few years, several RTX-family toxins, such as leukotoxins from *Actinobacillus* and *Mannheimia* species, and membrane microdomains have been reported to be closely related [18], [19]. Accordingly, we explored the possibility that the calcium influx induced by ACT could induce the internalisation of the toxin and/or of its receptor in macrophages, the integrin CD11b/CD18, and the role that raft-like membrane domains might have in such a process.

**Figure 1:**
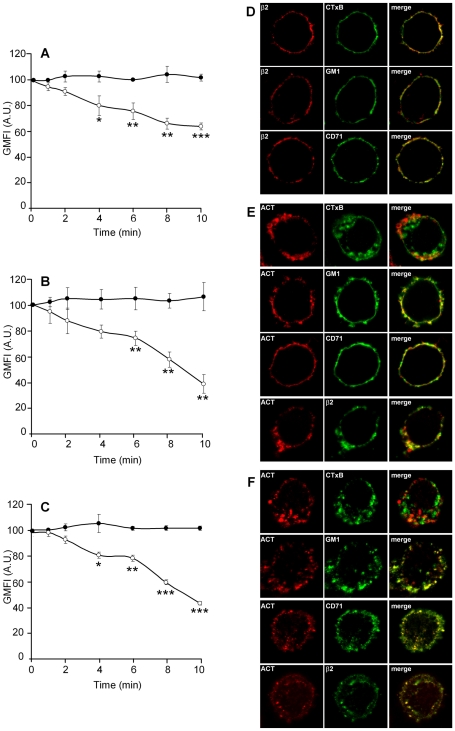
ACT is internalized and it triggers endocytosis of integrins and cholesterol-rich membrane microdomains in J774A.1 cells. Addition of ACT (35 nM) to J774A.1 cells results in time-dependent internalisation of ACT (A), GM1 (B) and the β2 integrin (C) ([•] control cells and [○] ACT-treated cells). Analysis by confocal microscopy of the localization of ACT, GM1 and the β2 integrin in J774A.1 cells treated for 2 min at 37°C with 35 nM toxin (D) and cells treated for 10 min at 37°C with the same toxin concentration (E). Internalisation in A, B and C was analysed with FACS as described in Materials and Methods. The data shown are the mean ± SEM of at least three independent experiments, with *p<0.05, **p<0.025 and ***p<0.001.

In J774A.1 macrophages incubated with ACT, the surface staining for the toxin, measured by flow cytometry, decreased in a few minutes (Fig. 1A). As antibodies cannot diffuse through the cell membrane, this finding is most readily explained by the endocytosis of ACT, suggesting that once bound to the cell, the toxin may be internalised. A raft marker phospholipid, the ganglioside GM1, and the integrin β_2_ seemed to follow the same fate as ACT, with a decrease in surface staining following similar kinetics, while with control cells the staining for these molecules did not change (Fig. 1 B and C, respectively). Furthermore, the internalisation process is dependent on ACT concentration (Figure S1). Putative shedding of Mac-1 molecules was ruled out as causative of the integrin epitope loss, as this phenomenon has not been reported for Mac-1 to date.

Confocal images of J774A.1 cells treated with ACT at 37°C for 2 minutes revealed more marked co-localization of the toxin with known raft-marker molecules, such as GM1 and the cholera toxin subunit B (CTxB), as well as with the integrin β_2,_ than with a non-raft marker, the transferrin receptor, CD71 (Fig. 1E). When longer incubation times were used (10 min) ACT, GM1 and the β_2_ integrin could be detected inside the cell (Fig. 1F), supporting the data obtained by flow cytometry, and demonstrating that ACT, GM1 and the β_2_ integrin are internalised. In untreated control cells, the β_2_ integrin co-localizes more abundantly with the non-raft marker CD71 than with the raft marker GM1 (Fig. 1D), in agreement with reports in the literature, i.e., that in non-stimulated macrophages the β_2_ integrin is mainly found in non-raft sites.

### Role of the ACT-induced calcium influx and importance of raft domains in the ACT-induced internalisation process

**Figure 2:**
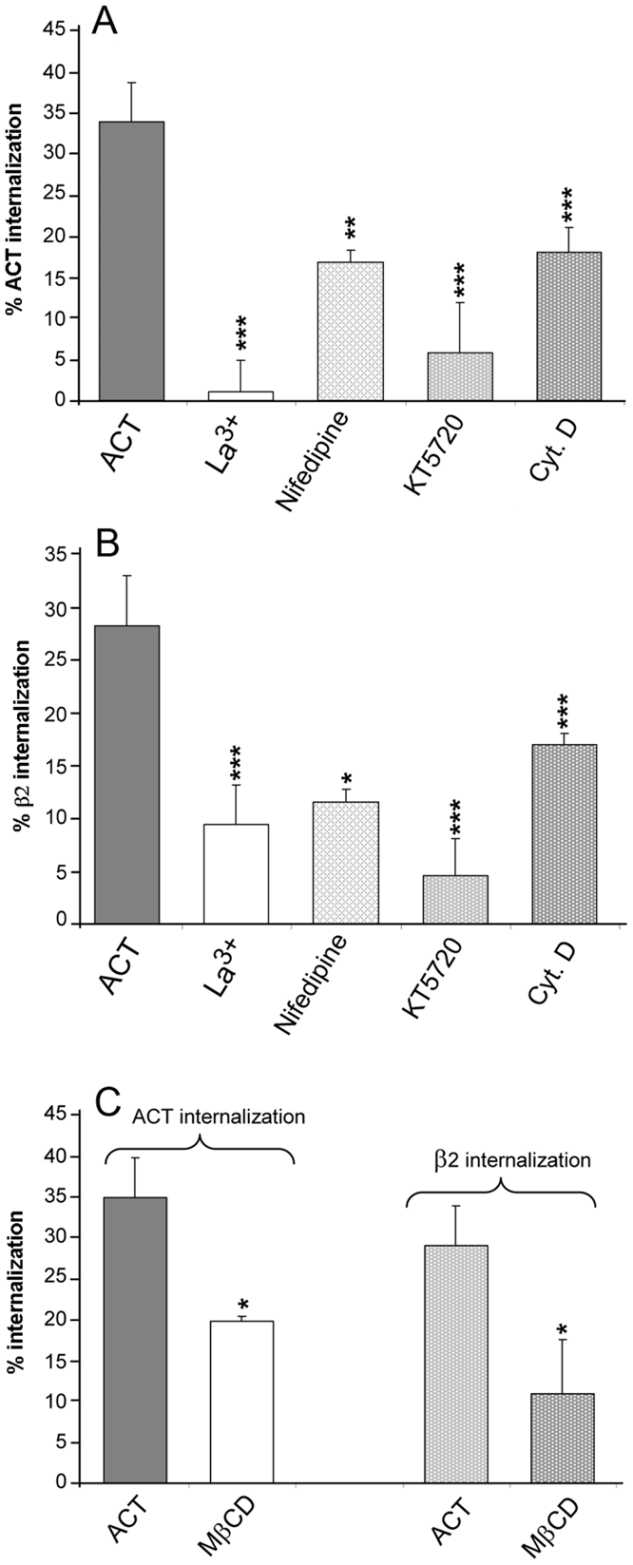
ACT-mediated endocytosis is dependent on Ca^2**+**^ influx, PKA activation, actin polymerization and raft-like microdomains integrity. J774A.1 macrophages were pre-incubated for 30 minutes at 37°C with inhibitors of the toxin-induced calcium influx, La^3+^ (100 µM) and nifedipine (10 µM), with an inhibitor of PKA, KT5720 (10 µM), and with an inhibitor of actin polymerization, cytochalasin D (10 µM), before the addition of ACT (35 nM). Then, the surface staining of ACT, GM1 and the β2 integrin was measured using a flow cytometer (panels A and B). The effect of cholesterol depletion was assayed by pre-incubation of cells with methyl-β-cyclodextrin (10 mM) for 30 minutes, after which cells were washed and then incubated with ACT (35 nM) (panel C). Internalisation was measured by FACS as described in Materials and Methods. Data shown are the mean ± SEM of at least three independent experiments, with *p<0.05, **p<0.025 and ***p<0.001.

Cell pre-incubation with La^3+^ (100 µM), a compound that has been shown (by us and others) to significantly inhibit the ACT-induced calcium influx [12], [13] prevented the decrease in surface staining of both ACT (Fig. 2A) and the integrin β_2_ (Fig. 2B). Pre-incubation of cells with nifedipine, a L-type calcium channel blocker (10 µM) and with KT5720, a cAMP-dependent PKA inhibitor (56 nM), also had a detectable effect on the surface staining of the two molecules (Fig. 2 A and B, respectively). These two compounds, nifedipine and KT5720, have previously been shown to considerably affect the calcium entry induced by ACT [13]. These results suggest that toxin-induced calcium influx is required for the internalisation process initiated by toxin binding.

In a previous publication from our laboratory we observed that both cAMP production and calcium influx induced by the toxin were affected by membrane cholesterol depletion [13]. Given this, we tested whether toxin-induced internalisation might also be affected by this compound.

We found that depletion of membrane cholesterol by pre-incubation of J774A.1 cells with 10 mM methyl-β-cyclodextrin β (MβCD), for 30 minutes (≈50% elimination of total cholesterol) before toxin addition, affected the degree of internalisation of ACT and the integrin to a comparable extent (Fig. 2C). This finding is consistent with a localization of the toxin in raft-like domains, demonstrating a greater sensitivity to be cholesterol-depleted by sterol sequestering agents, in comparison with the bulk lipid phase, and with the presence of raft marker molecules, such as GM1, co-localizing with ACT and that were also internalised together with the toxin itself (Fig. 1).

As negative control it was assayed the effect of the calcium entry inhibitors (La^3+^, nifedipine and KT5720) on the internalisation of a non-raft marker molecule such as the transferrin receptor. The results shown in Figure S2 support the notion that these compounds by themselves do not affect the cellular endocytic routes.

### Characterization of the ACT-induced internalisation: a clathrin-independent internalisation mechanism operates in parallel with clathrin-dependent endocytosis in ACT-treated cells

**Figure 3:**
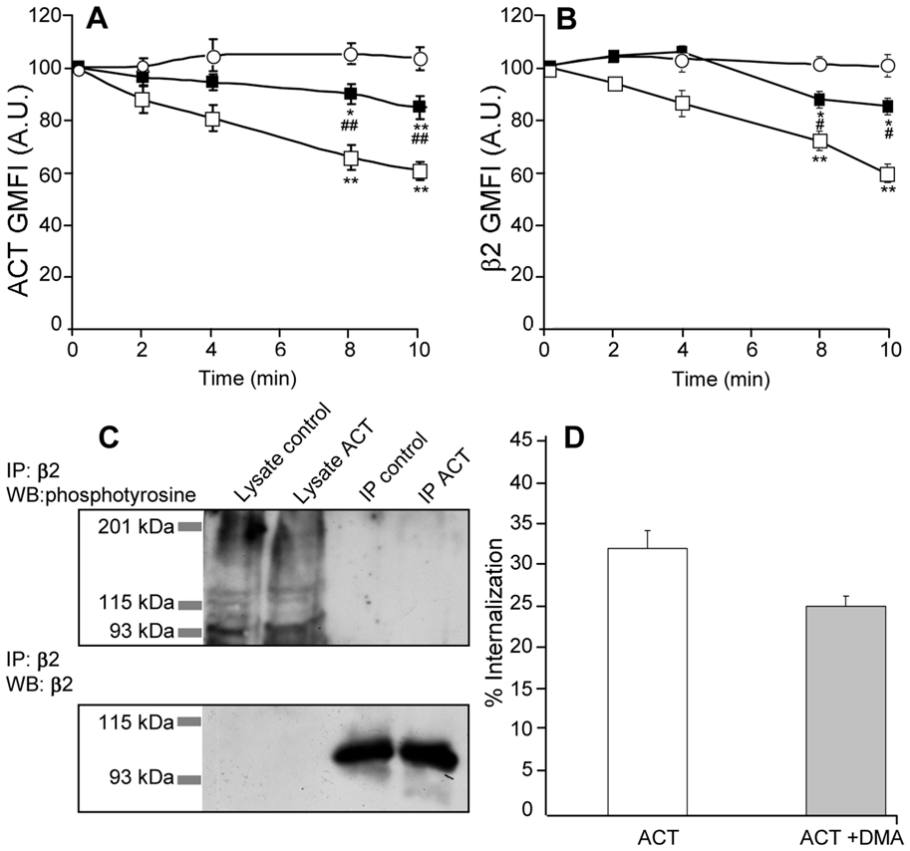
ACT internalisation takes place through two different routes of entry, likely acting in parallel . Cell-surface fluorescence of ACT and β2 integrin molecules in cells pre-treated for 30 min with chlorpromazine, an inhibitor of clathrin-mediated endocytosis, as followed by flow cytometry (A and B). ([○] control cells, 35 nM ACT-treated cells [□] and chlorpromazine-treated cells [▪]). In controls, cells were incubated for 2 min with ACT, then kept at 4°C to avoid any internalisation. Treatment of cells with 35 nM ACT does not induce integrin phosphorylation in tyrosine residues as assayed by Western blotting (C). Inhibition of macropinocytosis by DMA (200 µM) does not prevent ACT internalisation (D). FACS analysis of internalisation processes and immunoprecipitation were preformed as described in Materials and Methods. The data shown in (A), (B), (C) and (D) are the mean ± SEM of at least three independent experiments, with *p<0.05 and **p<0.025 with respect to control cells and #p<0.05 and ##p<0.025 with respect to ACT-treated cells.

In order to characterize the endocytic pathway followed by ACT we performed an internalisation assay in the presence of chlorpromazine. This compound is a specific inhibitor of clathrin-dependent receptor-mediated endocytosis. A dose-response curve was performed to establish the maximal inhibitory concentration of chlorpromazine (Figure S3). According to the obtained results we decide to use a 5 µg/ml chlorpromazine concentration throughout this experiment. In chlorpromazine-treated cells the internalisation of ACT and β2 was more modest, but still detectable (≈20%) (Fig. 3 A and B, respectively), suggesting that a clathrin-independent, ACT-induced internalisation mechanism might be operating in parallel in the ACT-treated cells. The relatively slow kinetics observed in this condition (Fig. 3 A and B, respectively) in comparison with the 40 to 180-second process seen in classical clathrin-mediated endocytosis of small ligands [32] seems consistent with this.

Ligand binding to CD11b/CD18 is known to induce activation of non-receptor tyrosine kinases that promote its phosphorylation, which in turn induces the internalisation of the integrin within clathrin-coated pits [33], [34]. Here, we did not detect tyrosine phosphorylation in the β_2_ chain of the integrin after immunoprecipitation of the integrin and Western blot analysis (Fig. 3C). Consistent with this result, genistein, a general inhibitor of tyrosine kinases was found not to significantly affect the internalisation of the integrin (data not shown).

To further elucidate the endocytic process involved in internalisation of the toxin, we tested dimethylamiloride (DMA) (200 µM) (Fig. 3D), a potent blocker of Na^+^/H^+^ exchange, which inhibits macropinocytosis. The presence of this compound did not significantly alter the toxin internalisation (Fig. 3D)****. In addition, Cav-1-mediated endocytosis was ruled out, as according to the literature this caveolae-forming protein is not expressed in J774A.1 macrophages [35].

### Internalisation of the α_M_β_2_ integrin in macrophages specifically depends on ACT-integrin interaction

Recruitment of ACT and its receptor, the α_M_β_2_ integrin, into a membrane raft location has been reported very recently, and was considered to be a direct consequence of the hydrolysis of cellular talin [30]. An ACT-induced intracellular [Ca^2+^] rise has been reported to activate calpain, a calcium-dependent cysteine protease, with concomitant talin hydrolysis and the release of integrins, which then move into raft domains [30].

Our results indicate that ACT and its integrin receptor are internalised from a raft-like membrane domain (Fig. 1 and Fig. 2C). According to the literature, however, the main site for the α_M_β_2_ integrin, in non-stimulated cells, is in a non-raft location. Therefore, we decided to explore whether ACT-β_2_ binding occurs outside the raft and then the complex becomes raft associated, inducing there signalling that then promotes the internalisation of the ACT-β_2_ complex-containing RLMD, or each molecule migrates independently, similarly encouraging RLMD internalisation.

**Figure 4:**
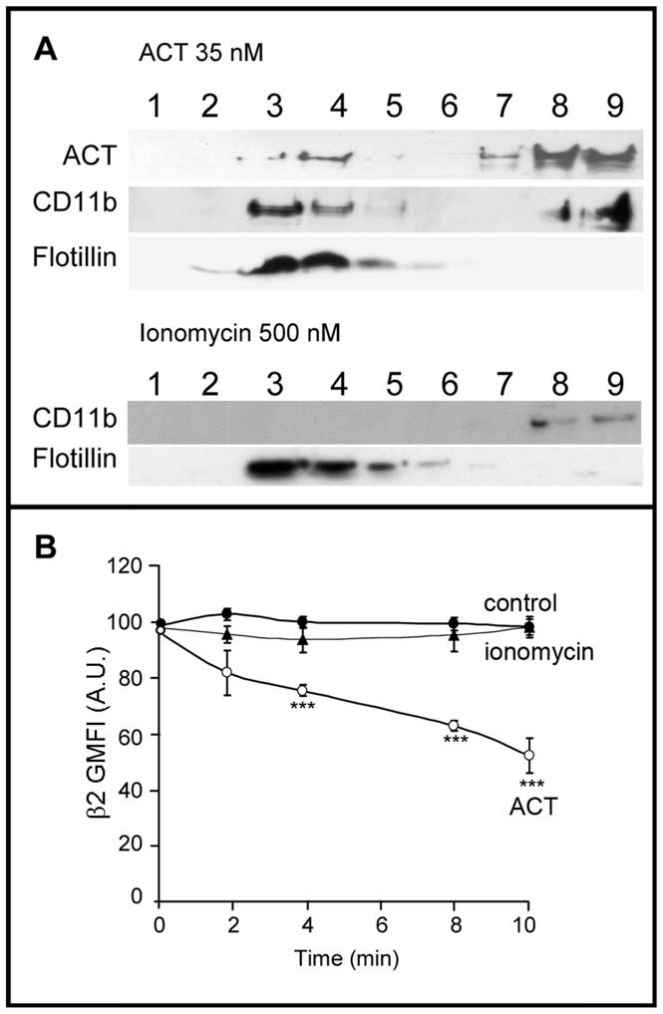
Endocytosis of the CD11b/CD18 integrin depends on ACT-receptor interaction and on ACT-induced calcium influx. Western blot of the fractions of a sucrose-density gradient stained with an anti-β2 integrin monoclonal antibody and with an anti-ACT monoclonal antibody, for ACT-treated cells (35 nM toxin) and for cells pre-incubated for 30 minutes at 37°C with 500 µM ionomycin before toxin addition. Flotillin, a raft marker protein, was used as control in membrane fractionation and gradient fraction analysis (A). Surface staining of -β2 integrin in control cells [•], ACT-treated cells (35 nM toxin) [○] and ionomycin-treated cells (500 µM ionomycin) [▴] (B). Membrane fractionation by sucrose gradient, analysis by immunoblotting and FACS were performed as described in Materials and Methods. The data shown in (A) are representative of a set of three independent experiments and the data shown in (B) are the mean ± SEM of at least three independent experiments, with ***p<0.001 with respect to control cells.

To address this question we tested whether it was possible to induce migration of “empty” β_2_ integrin molecules into microdomains, by treating J774A.1 cells with a calcium ionophore, ionomycin, in the absence of the toxin. This approach was based on the assumption that if migration of the β_2_ integrin molecules depends only on their calpain-mediated release from the talin cytoskeleton, then it would be expected that the induction of an artificial intracellular [Ca^2+^] rise by the calcium ionophore could enable the migration of integrin molecules into rafts. Results are shown from a representative experiment using Western blot analysis of SDS-PAGE gels loaded with fractions from a sucrose-density gradient of cells treated with ionomycin (Fig. 4A). No protein band corresponding to the CD11b/CD18 integrin was detectable in the upper fractions of the sucrose gradient. In addition, flow cytometry was performed to test whether internalisation of the β_2_ integrin occurred in ionomycin-treated cells (Fig. 4B). When J774A.1 macrophages in suspension were treated with 500 nM ionomycin, no decrease of the integrin surface signal was detected. Both results suggest that the β_2_ integrin needs to be in a complex with ACT in order to be located in raft-like domains and be internalised.

### Signalling partners in the ACT-induced integrin internalisation

**Figure 5:**
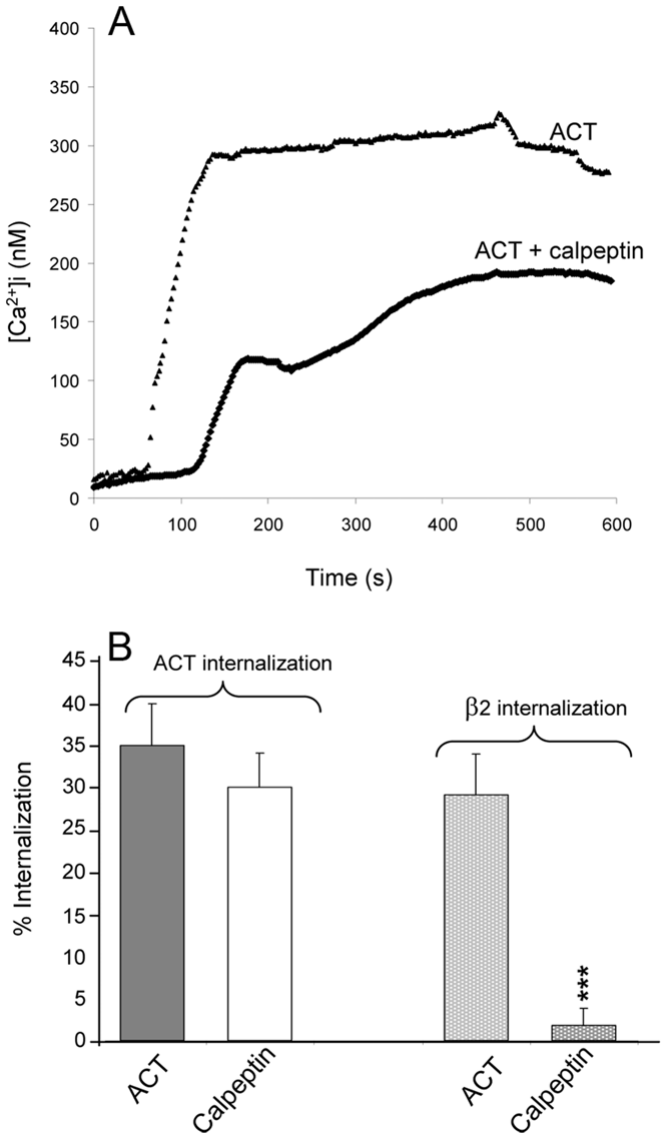
Calpain is involved in the endocytosis triggered by ACT . Cell pre-incubation with calpeptin (50 µM) inhibits calcium influx mediated by ACT (A) and the internalisation of the integrins (B). Internalisation and Ca^2+^ influx were determined as described in Materials and Methods. Data shown are the mean ± SEM of at least three independent experiments, with *p<0.05 and ***p<0.001.

Very recently, calpeptin, a calpain inhibitor, was reported to block talin hydrolysis, to strongly inhibit the association of ACT with DRMs (detergent resistant membranes) and to decrease by at least a factor of two the capacity of cell-associated ACT to translocate the AC enzyme [30]. Contrary to these results reported by Bumba et al. [30], we found that pre-incubation of cells with 100 µM calpeptin markedly affected the ACT-induced calcium influx, with a substantial effect on the kinetics of the cation entry, which now showed a lag period of around 100 seconds and a lower amplitude (Fig. 5A). Production of cAMP by the toxin was also affected by treatment with calpeptin (data not shown) in agreement with the findings of the aforementioned authors [30].

The effect of calpeptin on ACT-induced internalisation in macrophages was assayed by flow cytometry (Fig. 5B). This compound nearly completely abrogated the internalisation of the CD11b integrin. Its effect on the toxin internalisation was not however so prominent, suggesting that the toxin has dual processes for internalisation, one associated with its receptor and another independent of it. This is in agreement with the aforementioned finding that a clathrin-insensitive entry pathway also operates in the internalisation of ACT.

In addition, we tested the occurrence of ACT internalisation in CD11b^−^ cells, i.e., cells that do not naturally express the CD11b/CD18 toxin receptor. Flow cytometry experiments showed very modest, but reproducible, internalisation of the toxin in ACT-treated CHO cells (Figure S4B). Images taken using a confocal microscope showed co-localisation of ACT with Cav-1 after 2 minutes of incubation with ACT (Figure S4B) supporting the aforementioned results and indicating that ACT may be internalised in the absence of the integrin receptor.

### Integrin removal from the cell surface decreases macrophage adhesion capacity

**Figure 6:**
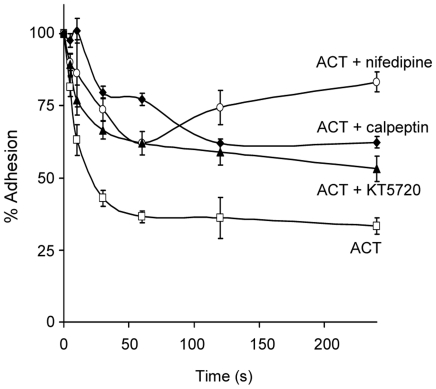
ACT-induced decrease of macrophage adhesion. Treatment with 35 nM ACT for 10 min reduces J774A.1 cell adhesion. The effect of the toxin on adhesion was partially reduced by pre-treatment with nifedipine (100 µM), a Ca^2+^ channel inhibitor, KT5720 (56 nM), a PKA inhibitor and with calpeptin (50 µM), an inhibitor of the protease calpain. All molecules were pre-incubated for 30 min with J774A.1 cells before the addition of ACT. Adhesion was measured as described in Materials and Methods; the data shown is the mean ± SEM of at least three independent experiments.

Integrins participate in cellular functions such as cell-cell or cell-extracellular matrix adhesion that are essential in the immune response [36]. In particular, β2 integrins are predominantly responsible for firm cellular adhesion during processes such as diapedesis and extravasation, phagocytosis, and cell locomotion. We found that treatment of J774A.1 cells for 10 min at 37°C with ACT reduced the capacity of these cells to attach to fibrinogen-coated plates by around 50% (Fig. 6). The inhibition of toxin-induced calcium influx by nifedipine (10 µM) and KT5720 (56 nM) managed to inhibit, at least partially, their adhesion (Fig. 6). Such a loss of adhesiveness is a logical result of the decreased number of integrin molecules on the cell surface due to internalisation of membrane domains containing such molecules. ACT-induced calcium-influx is required for this effect. On other hand, we also observed that pre-incubation of cells with calpeptin, an inhibitor of calpain, produced a marked recovery in the ability of the macrophages to adhere to fibrinogen, suggesting that integrin molecules have to be released from the cytoskeletal talin to be internalised.

## Discussion

ACT is a key virulence factor secreted by the whooping cough bacterium *B. pertussis*. The toxin belongs to the so-called RTX family of bacterial proteins that share several features, including a dedicated secretion mechanism, and a strict calcium-binding and post-translational fatty-acylation requirement to exhibit full biological activity. From recent experiments, it may be concluded that two additional features are also shared by members of this family: the induction of rises in intracellular calcium and the involvement of cholesterol-rich membrane domains, at some (maybe several different) stages, in their toxicity mechanisms [13], [19], [30], [37]–[39].

[Ca ^2+^]_i_ regulates important cellular pathways, including those leading to cell death by apoptosis or necrosis, therefore, modulation of [Ca ^2+^]_i_ constitutes one of the most widely used cell signalling mechanisms. Indeed, [Ca ^2+^]_i_ is tightly controlled and must be maintained at a low level (≤1 µM), as high concentrations for prolonged periods have a lethal effect on most types of cell. The elevation and modulation of free cytosolic calcium concentrations by bacterial toxins has been described as one of the basic strategies of host cell manipulation used by pathogens [14].

Numerous pathogenic bacteria, bacterial toxins or viruses have been reported to use rafts or raft-like membrane domains as cell surface platforms to interact, bind and possibly enter host cells. Bacteria can induce Ca^2+^ responses that play a role in the cytoskeletal rearrangements required for cell binding and for internalisation of the microorganism [14].

Here we report evidence that ACT and integrin molecules, along with other raft components, are rapidly internalized by macrophages in a toxin-induced calcium rise-dependent process (Figs. 1, 2 and 3). This toxin-triggered internalisation occurs through two different routes of entry, chlorpromazine-sensitive receptor-mediated endocytosis and clathrin-independent endocytosis, which maybe act in parallel.

We have found that toxin internalisation from raft domains also occurs in cells devoid of receptors, in a toxin-induced calcium-rise dependent manner, thus suggesting that the process may occur independently of the presence of the β_2_ integrin (Supplementary data). ACT binding and clustering in patches within membrane microdomains has previously been reported in other cells, such as erythrocytes, which do not contain the integrin receptor [40]. On the contrary, as judged by the results shown in Fig. 4, the CD11b/CD18 integrin seems to require the formation of a “complex” with the toxin, and not only an increase in intracellular calcium, to move into raft-like membrane domains or to form molecular clusters. This might be due to the fact that calcium elevation induced by the ionophore does not occur locally, but rather throughout the sub-membrane. In addition, the toxin-receptor interaction may induce some kind of signalling required for the process. Bumba et al. [30] have very recently reported that ACT and the α_M_β_2_ receptor relocate together into DRMs. An intriguing finding in this same paper is that upon permeabilisation of cells for extracellular calcium with ionomycin (500 nM), up to 15% of a mutant toxin (ACT E570K+E581P), unable on its own to induce any calcium entry and thus DRM relocation, was found to be associated with DRMs. For another RTX toxin, *Actinobacillus actinomycetemcomitans* leukotoxin (Ltx) it was recently shown that cells that do not express lymphocyte-associated antigen-1 (LFA-1) receptor molecules failed to accumulate Ltx in their raft fractions [18], suggesting that the association of Ltx and LFA-1 within lipid rafts was essential for Ltx to be in rafts. However, in the same paper, toxin-induced clustering of both receptor (LFA-1) and non-receptor integrins (α_4_β_1_) was detected, indicating that the integrin mobilisation was not the result of a toxin-LFA-1 interaction, but possibly due to the toxin-induced [Ca^2+^] increase [18].

In our work, we have found that the two toxin activities ACT-induced calcium influx and toxin-induced internalisation are markedly affected by cholesterol depletion (Fig. 5). Though the direct participation of cholesterol-enriched membrane rafts in these two particular processes is indeed one of the most plausible possibilities, it must not be forgotten that cholesterol may influence multiple processes in a cell. The cholesterol effect is evident both in receptor-expressing cells and in CD11b^−^ cells, underlining the importance of the membrane properties in ACT action. Involvement of cholesterol-enriched domains in several steps of ACT activity has been reported in recent years by various different groups. Decreased ACT binding and clustering after cholesterol removal from erythrocytes was reported by Votjova et al. [40]. Modulation of the ACT-induced solute efflux by cholesterol content was reported in artificial membranes [11]. Translocation of the AC domain and subsequent cAMP accumulation has also been found to be impaired by cholesterol depleting compounds [13], [30]. On the other hand, a variety of ion channels, including members of all major ion channel families, have been shown to be regulated by changes in the level of membrane cholesterol and partition into cholesterol-rich membrane domains [41]. Moderate depletion of plasma membrane cholesterol has been reported to have inhibitory effects on chlatrin-independent endocytosis, and also in some cases, on chlatrin-mediated endocytosis [42]. Therefore, it seems to be proving difficult to identify in which particular step cholesterol elimination is being more detrimental, or its enrichment more necessary, for ACT activity.

It is expected that multiple cell-types will be exposed to ACT during infection with *B. pertussis*. Some of them, such as mucosal epithelial cells do not express the receptor CD11b/CD18, but respond, in a specific manner, to nM concentrations of ACT [43]. While CD11b/CD18 expression greatly augments the intoxication of cells by ACT [7], it is clearly not required for effective intoxication [9], [43]–[45]. This certainly suggests that additional factors, aside from the receptor molecule, might also be of great importance in the overall mechanism of action of ACT. In view of the results reported here, and those previously published by other groups, there is no doubt that target cell membrane properties are much more relevant in ACT activity than previously thought.

An interesting observation that may contribute to improving our understanding of the downstream effects of calcium is the effect of calpeptin on ACT-treated cells. This calpain inhibitor affects the toxin-induced calcium influx, the toxin production of cAMP and the internalisation of the integrin molecules (Fig. 5). The questions are, how are these three effects related? Which is the role of talin in the endocytosis of integrin? is clustering of integrins required for endocytosis? Experiments have been conducted in our laboratory to attempt to provide an answer (manuscript in preparation).

Our results strongly point towards an intrinsic ability of ACT to, directly and efficiently, insert into raft domains triggering there a range of different effects, without a strict requirement for receptors. Given this feature, ACT might be considered a “*raftophilic*” protein, a termed previously used to describe the tendency shown by certain lipids and proteins to preferably partition into liquid-ordered membrane domains [46].

Another important finding in this work has been the observation that the removal from the cell surface of β_2_ integrin molecules by ACT action significantly decreases macrophage adhesion capacity. Integrins have many essential functions in the immune response such as adhesion and phagocytosis [47]. Specifically, integrins are involved in cell adhesion to the extracellular matrix and to other cells, regulating leukocyte extravasation during inflammation, the initiation of the immune response and leukocyte traffic under normal physiological conditions. Many of these processes are very important in the host immune response against any bacterial infection. Impairment of this response by the removal of domains that contain key molecules such as integrins, and perhaps other important signalling molecules, from leukocyte plasma membrane may represent a beneficial strategy followed by pathogenic *Bordetella pertussis* to circumvent the host immune system.

As a corollary from all the data, we conclude that the plasma membrane physicochemical properties undoubtedly play a key role in the mechanism of action of ACT, and very likely, other RTX toxins. In this regard, many questions remain to be explored in the near future: Does ACT exploit or depend on the inherent properties of cholesterol-enriched domains to exert its cytotoxic activities? Is the toxin itself able to locally alter the physical properties of the lipid bilayer? And could ACT even indirectly induce local changes in the membrane lipid composition, i.e., activating calcium-dependent sphingomyelinases? Experiments are currently ongoing in our laboratory to provide answers.

## Materials and Methods

### Antibodies and reagents

Anti-RTX was from Santa Cruz Biotechnology (Santa Cruz, CA, USA); anti-talin, anti-flotillin-1, anti-caveolin-1, La^3+^, Cytochalasin D, methyl-β-cyclodextrin, nifidepine, chlorpromazine, DMA, ionomycin and genistein from Sigma(St Louis, MI); anti CD11b, anti-β2 and anti-GM1 from abcam (Cambridge, UK); calpeptin and KT5620 from Calbiochem (Merk, Germany); anti-mouse Texas Red, anti-rabbit FITC and anti-goat Alexa Fluor® 594 from Invitrogen, Molecular Probes(Carlsbad, CA).

### ACT purification

ACT was expressed in *Escherichia coli* XL-1 (Stratagene) blue cells transformed with pT7CACT plasmid and purified as previously described [10].

### Cell culture

J774A.1 murine macrophages (ATTC, number TIB-67) and CHO cells (ATTC, number CCL-61) were cultured at 37°C in DMEM supplemented with 10% (v/v) heat-inactivated foetal bovine serum, 100 U/ml penicillin, 100 µg/ml streptomycin and 4 mM L-glutamine in a humidified atmosphere with 5% CO_2_.

### Flow cytometry

To determine the internalisation kinetics of integrins, GM1 and ACT, macrophages were incubated with 35 nM ACT for 2 min at 37°C allowing the toxin to bind with no time enough to allow internalisation. Then, cells were washed 3 times with ice-cold PBS to remove unbound toxin, and incubated again at 37°C for various intervals (0, 2, 4, 6, 8 and 10 min). Cells were then washed, detached in ice-cold PBS, labelled under non-permeabilizing conditions against β2 integrins, ACT, or GM1 with the primary antibodies for 1 h at 4°C, then centrifuged, washed and mixed in buffer containing FITC-conjugated secondary antibodies and incubated for 1 h at 4°C. Cells were then washed, mixed in ice-cold PBS and analyzed in a FACSCalibur flow cytometer (Beckton Dickinson). Geometric MFI data was used to calculate the percentage of internalisation at each point, which is expressed as the percentage of fluorescence of the cells at each point of the kinetic relative to the total fluorescence of the cells obtained after toxin binding for 2 min.

Total cell surface bounded ACT at 4°C, and the internalisation kinetics at 37°C in CHO cells were determined in the same conditions as described above.

### Confocal microscopy

Cells were grown to sub-confluency onto 12 mm diameter glass coverslips placed into the wells of a 24-well plate. 35 nM ACT was added to the medium and cells were incubated for 2 or 10 min. Then, treated cells were washed three times in PBS to remove unbound toxin, fixed for 10 min with 3.7% paraformaldehyde and permeabilized with acetone at –20°C. Control cells followed the same procedure. Then samples were incubated with the appropriate primary antibodies for 1 h followed by incubation with Texas Red- or FITC-conjugated secondary antibodies. Coverslips were mounted on a glass slide and samples were visualized using a confocal microscope (Olympus IX 81) with sequential excitation and capture image acquisition with a digital camera (Axiocam NRc5, Zeiss). Images were processed with Fluoview v.50 software.

### Immunoprecipitation of β2 integrin

Cells treated for 10 min with ACT were homogenized in lysis buffer (20 mM Tris-HCl [pH 7.65], 1 mM sodium orthovanadate, 2% Triton X-100, 100 µg of aprotinin per ml, 100 µg of leupeptin per ml, 10 µg of pepstatin per ml, and 2 mM phenylmethylsulfonyl fluoride). Lysates were incubated with anti-β2 antibody overnight at 4°C. Sepharose protein G beads (20 µL) were added to the lysates and the mixture was incubated for 4 h at 4°C. After incubation, the mixture was centrifuged and the supernatant was discarded. The pellet was washed three times with 200 µl of wash buffer, 50 µl of elution buffer was added, and the suspension was vortexed gently for 30 s and pelleted by centrifugation. Aliquots (25 µl) of 2× SDS-PAGE loading buffer (without 2-mercaptoethanol) were added to each tube containing the immunoprecipitated proteins (pellet), boiled for 4 min, loaded, and resolved on 8.5% SDS gels. Separated proteins were transferred onto a nitrocellulose membrane and subjected to Western blotting.

### Isolation and analysis of DRMs

DRMs from J774A.1 cells were prepared according to Olsson and Sundler [48]. Briefly, control and 35 nM ACT-treated cells were lyzed in 10 mM Tris-HCl, 200 mM NaCl, 1 mM EDTA pH 7.4, containing 1% (v/v) Triton X-100, for 30 minutes at 4°C. The extracts were brought to 40% sucrose and placed at the bottom of 5–35% sucrose gradient with 1% TX-100. Gradients were ultracentrifuged for 18 hours at 306,000×g at 4°C. Fractions of 1 ml were harvested from the top of the gradient and stored at −20°C. Equal amounts of protein from each fraction were analysed by SDS-PAGE and western blott, and protein bands on X-ray film were quantified by densitometric scanning using a Bio-Rad Imaging densitometer.

### Western blotting

Proteins were separated electrophoretically on a 8.5% SDS-polyacrylamide gel and transferred to nitrocellulose membrane. The membranes were then blocked overnight at 4°C, and after 2 h of incubation with the corresponding primary antibodies, membranes were washed and exposed to the secondary antibodies for 1 h at room temperature. Proteins were detected using the enhanced chemiluminiscence detection system (ECL®, Amersham Biosciences). The Quantity One® Image Analyzer software program (Bio-Rad) was used for quantitative densitometric analysis.

### Measurements of intracellular [Ca^2+^]

Calcium influx into J774A.1 cells was measured as previously described [10]. Briefly, J774A.1 cells grown on glass coverslips, were loaded with 2 µM fura2-AM for 30-45 min. The coverslips were mounted on a termostatized perfusion chamber on a Nikon Eclipse TE 300 based microspectofluorometer and visualized with a ×40 oil-immersion fluorescence objective lens. At the indicated time, 35 nM ACT was added and the intracellular Ca^2+^ levels were determined using the method of Grynkiewicz et al. [49]. The ratio of light exited at 340 nm to that at 380 was determined with a Delta-Ram system (Photon Technologies International, Princeton).

### Adhesion assay

50×10^3^ cells were incubated 10 min with 35 nM ACT then, cells were washed and distributed in fibronogen coated 96-well plates. Cells were subsequently incubated up to 4 h at 37°C, with 5% CO_2_. At different times, released or came off cells were gently removed by pipette and the attached cells were gently washed, fixed for 15 min with 0.25% glutaraldehyde, stained for 30 min with 1% crystal violet, washed several times and incubated for 15 min at room temperature with 15% acetic acid. The staining intensity corresponding to the amount of attached cells was measured in a photometer at 595 nm.

### Statistical analysis

All measurements were performed at least 3 times, and results are presented as mean ± s.d. Levels of significance were determined by a two-tailed Student's *t*-test, and a confidence level of greater than 95% (p<0.05) was used to establish statistical significance.

## Supporting Information

Figure S1 **Dose-response curve for the internalisation of ACT, β2 integrin and GM1 molecules.** Addition of ACT to J774A.1 cells at the concentrations indicated in the figure results in a toxin concentration-dependent internalisation of ACT, β2 integrin and GM1. Internalisation after 10 min of incubation with the toxin was analysed with FACS as described in Materials and Methods. The data shown are the mean ± SEM of three independent experiments.
(TIF)

Figure S2 **Effect of several inhibitors on the internalisation of transferrin in toxin-untreated cells.** Treatments of cells with with La^3+^, nifedipine, KT5720 or cytochalasin D do not affect by themselves the endocytosis of FITC-labelled transferrin. FACS analysis of internalisation was preformed as described in Materials and Methods. The data shown are the mean ± SEM of three independent experiments.
(TIF)

Figure S3 **Dose-response curve to establish the maximal inhibitory concentration of chlorpromazine.** Internalisation of FITC-labelled transferrin or of ACT was measured in the absence or presence of the indicated chlorpromazine concentrations (A and B). Cells were incubated with FITC-labelled transferrin or with ACT for 10 min in the presence of the inhibitor at the concentrations indicated in the figure and FACS analysis of internalisation was preformed as described in Materials and Methods. The data shown are the mean ± SEM of three independent experiments.
(TIF)

Figure S4 **ACT internalizes and co-localizes with Cav-1 in CHO cells.** Addition of ACT (35 nM) to CHO cells results in co-localization of ACT with Cav-1 (A) and in a time-dependent internalisation of ACT (B) ([•] control cells and [○] ACT-treated cells). Confocal microscopy analysis of CHO cells and internalisation were analysed as described in Materials and Methods. Briefly, quantification of ACT in control cells was performed after very short cell incubation with the toxin (2 min). To avoid toxin internalisation cells were kept at 4°C. This allows the assignment of 100% of the ACT bound to the membrane. The data shown are the mean ± SEM of at least three independent experiments, with **p<0.025 and ***p<0.001.
(TIF)
