# Supplementary figures and images for: Adenylate Cyclase Toxin Promotes Internalisation of Integrins and Raft Components and Decreases Macrophage Adhesion Capacity
Source: PLoS One. 2011 Feb 23;6(2):e17383. doi: 10.1371/journal.pone.0017383 (PMC3044178; doi:10.1371/journal.pone.0017383)

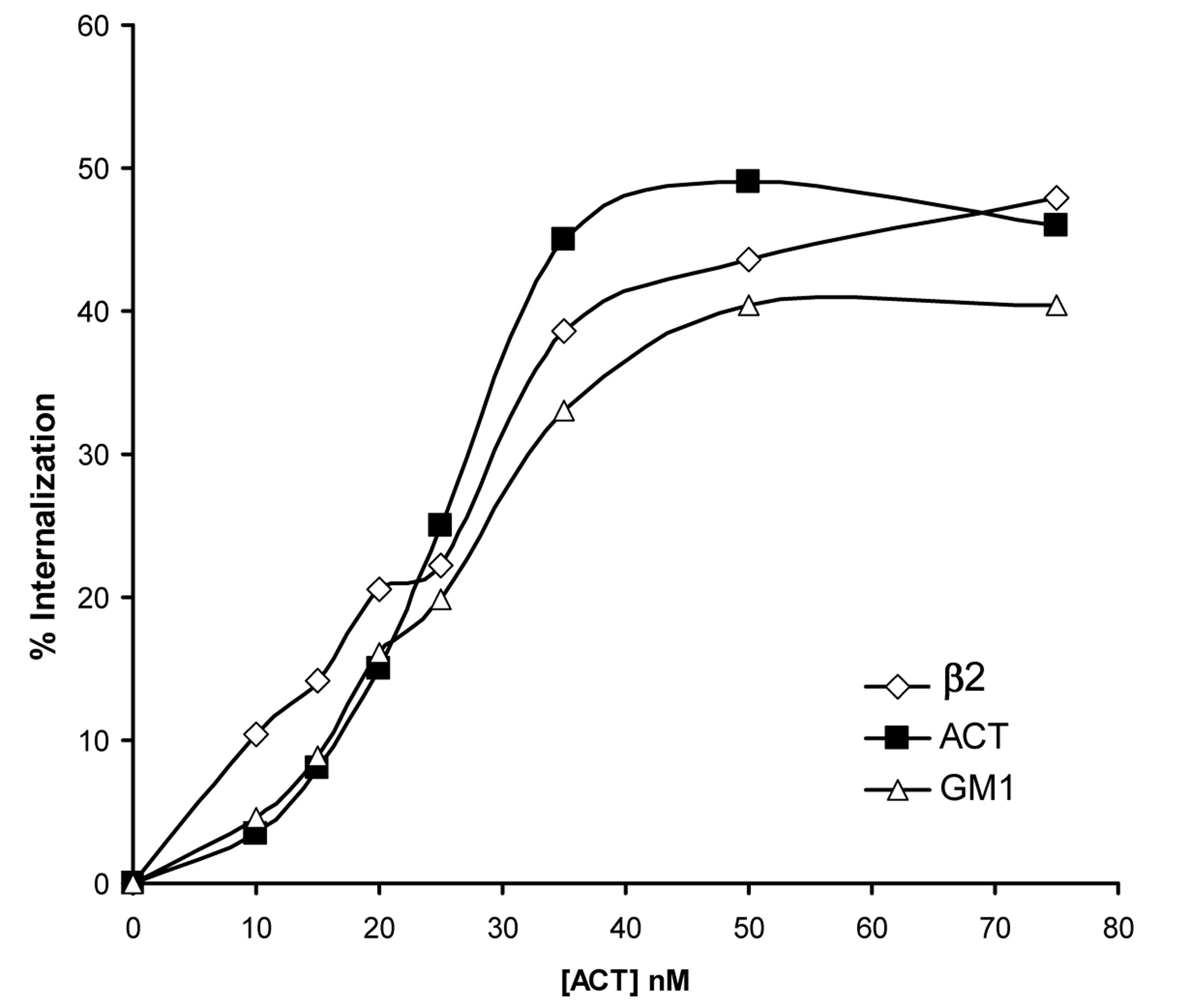

Supplement: Figure S1 — Dose-response curve for the internalisation of ACT, β2 integrin and GM1 molecules. Addition of ACT to J774A.1 cells at the concentrations indicated in the figure results in a toxin concentration-dependent internalisation of ACT, β2 integrin and GM1. Internalisation after 10 min of incubation with the toxin was analysed with FACS as described in Materials and Methods. The data shown are the mean ± SEM of three independent experiments. (TIF) [file pone.0017383.s001.tif]

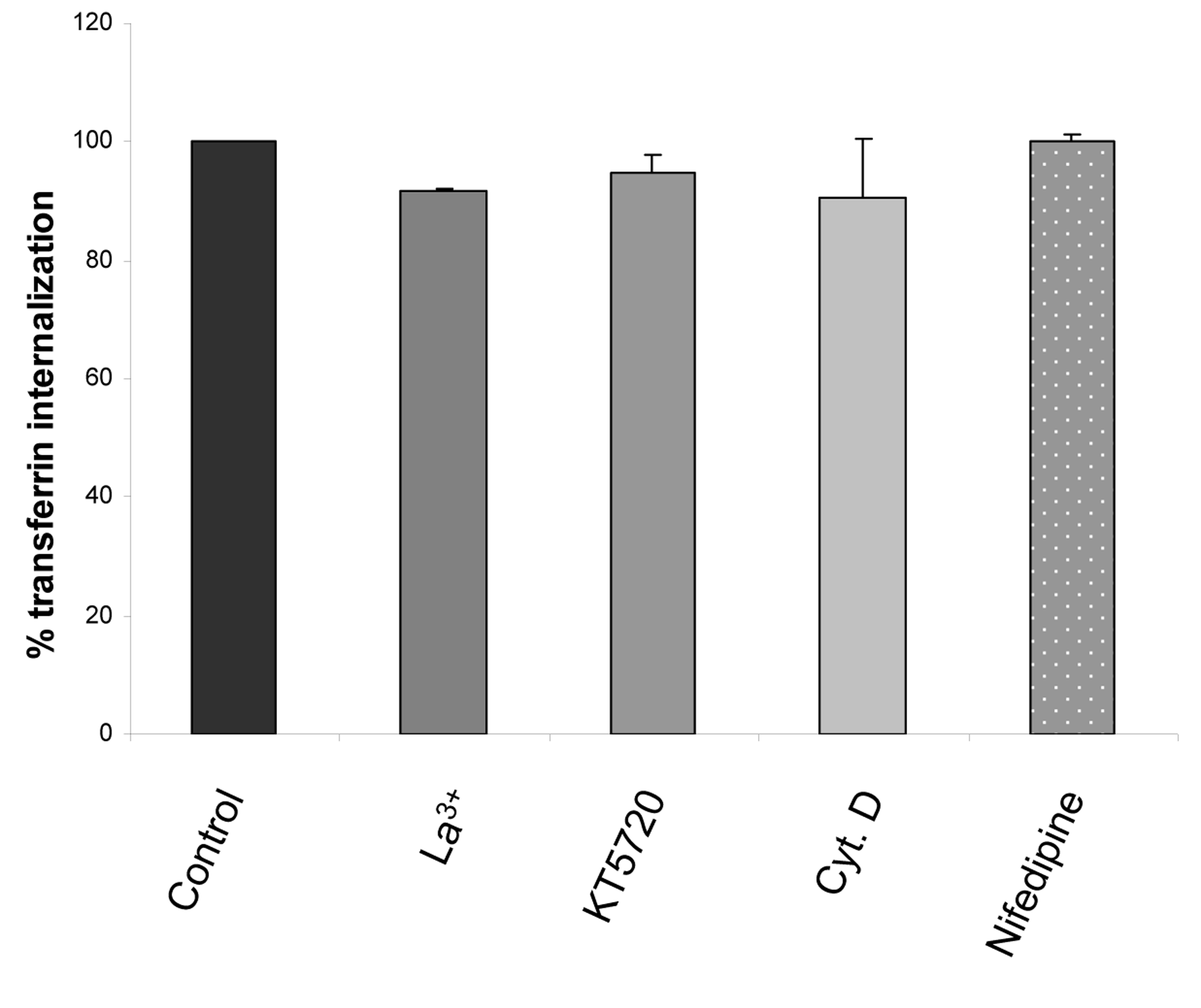

Supplement: Figure S2 — Effect of several inhibitors on the internalisation of transferrin in toxin-untreated cells. Treatments of cells with with La3+, nifedipine, KT5720 or cytochalasin D do not affect by themselves the endocytosis of FITC-labelled transferrin. FACS analysis of internalisation was preformed as described in Materials and Methods. The data shown are the mean ± SEM of three independent experiments. (TIF) [file pone.0017383.s002.tif]

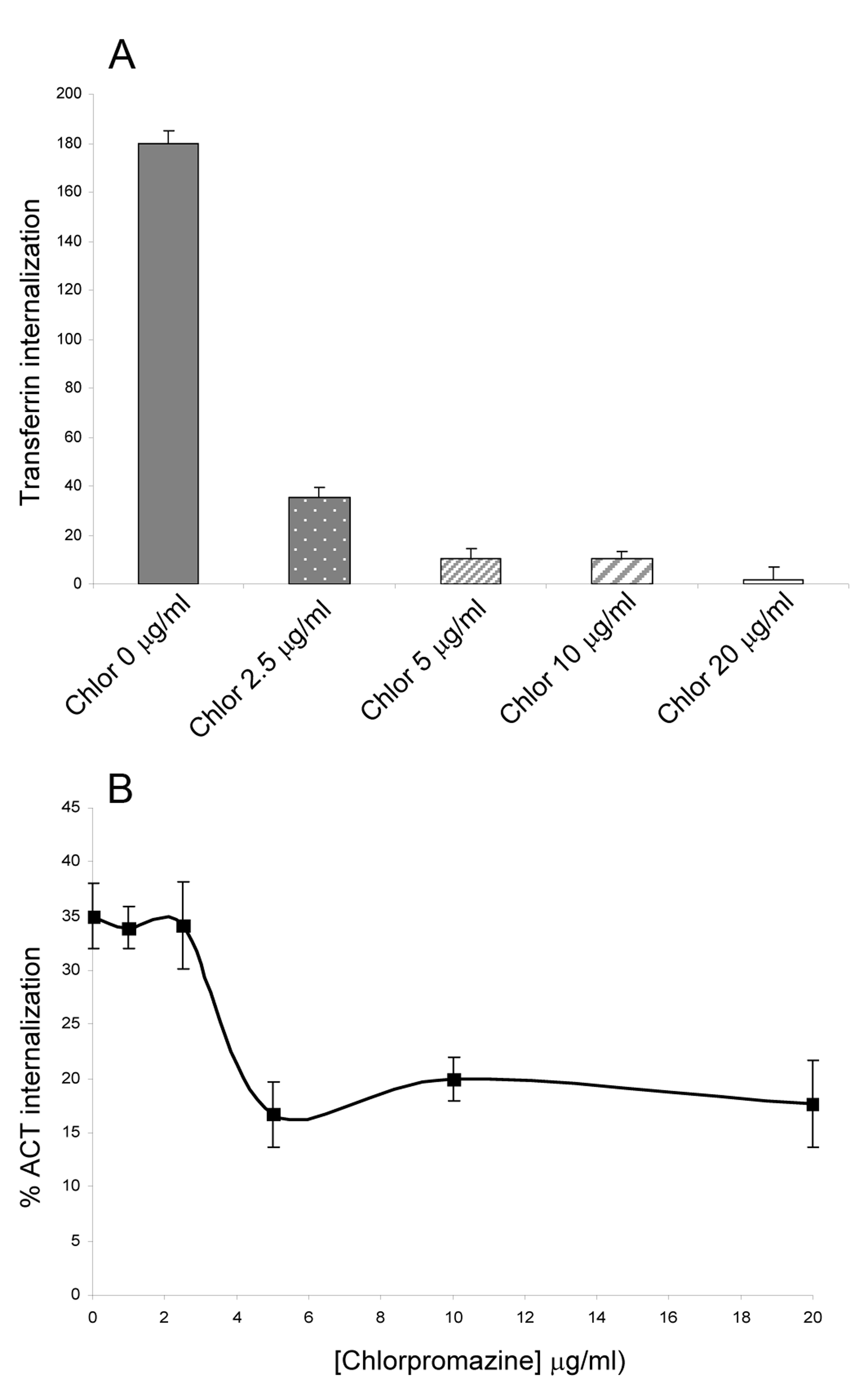

Supplement: Figure S3 — Dose-response curve to establish the maximal inhibitory concentration of chlorpromazine. Internalisation of FITC-labelled transferrin or of ACT was measured in the absence or presence of the indicated chlorpromazine concentrations (A and B). Cells were incubated with FITC-labelled transferrin or with ACT for 10 min in the presence of the inhibitor at the concentrations indicated in the figure and FACS analysis of internalisation was preformed as described in Materials and Methods. The data shown are the mean ± SEM of three independent experiments. (TIF) [file pone.0017383.s003.tif]

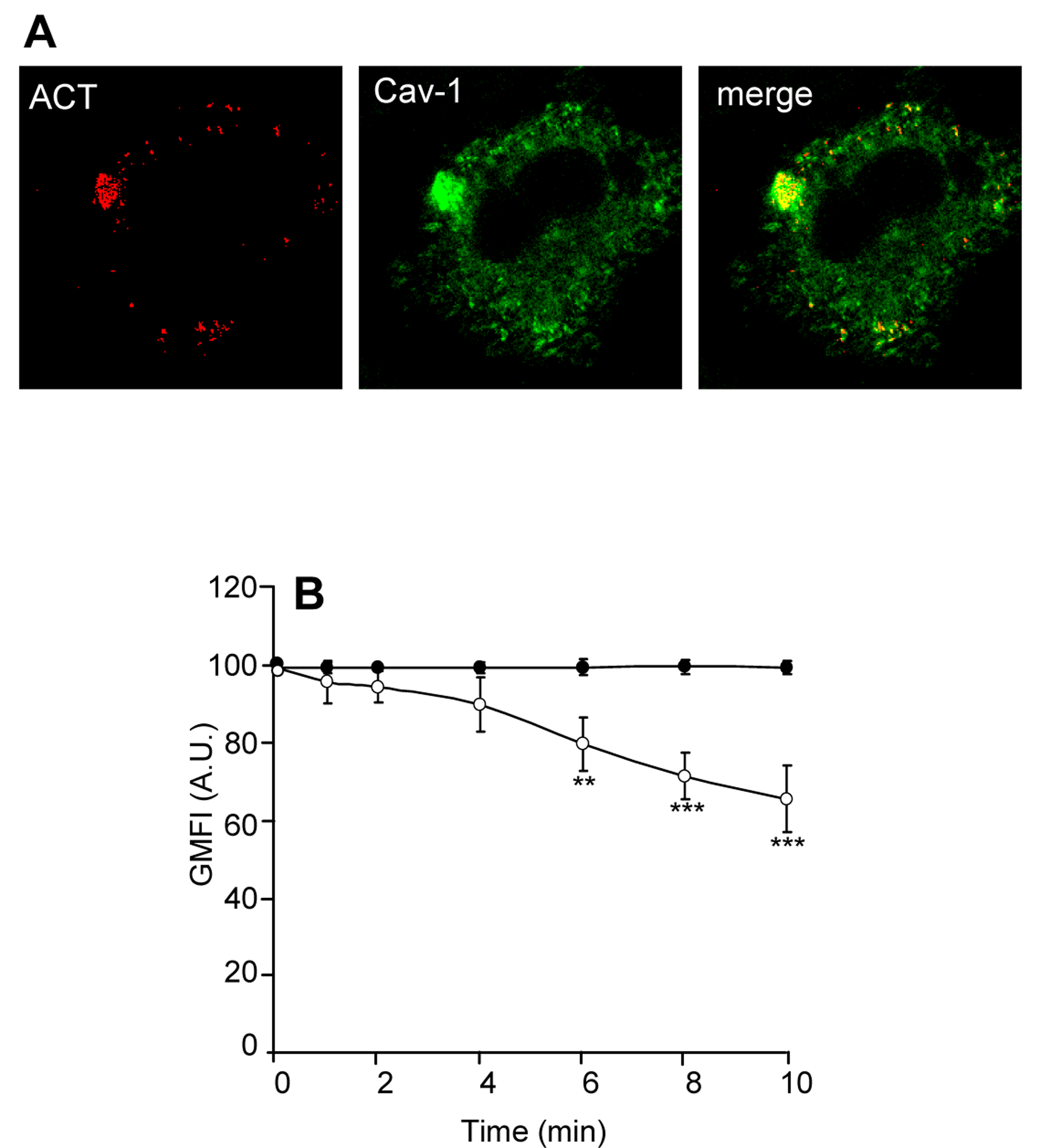

Supplement: Figure S4 — ACT internalizes and co-localizes with Cav-1 in CHO cells. Addition of ACT (35 nM) to CHO cells results in co-localization of ACT with Cav-1 (A) and in a time-dependent internalisation of ACT (B) ([•] control cells and [○] ACT-treated cells). Confocal microscopy analysis of CHO cells and internalisation were analysed as described in Materials and Methods. Briefly, quantification of ACT in control cells was performed after very short cell incubation with the toxin (2 min). To avoid toxin internalisation cells were kept at 4°C. This allows the assignment of 100% of the ACT bound to the membrane. The data shown are the mean ± SEM of at least three independent experiments, with **p<0.025 and ***p<0.001. (TIF) [file pone.0017383.s004.tif]
